# Dependence-related screening positivity among regular stimulant-laxative users with chronic constipation: a multicenter prospective observational study

**DOI:** 10.1007/s00535-026-02455-9

**Published:** 2026-05-29

**Authors:** Yo Ishihara, Eikichi Ihara, Mitsuru Esaki, Kota Takahashi, Naoki Tomita, Thanh Huy Dang, Mayumi Ohira, Mayu Horiuchi, Ayaka Uchida, Yukiko Toriumi, Toshihiro Nishizawa, Hidenori Ohkubo, Kento Imajo, Shunsuke Oyamada, Tomoyuki Iwasaki, Takeo Kurihashi, Atsushi Yamauchi, Michiko Nakazato, Atsushi Nakajima, Takaomi Kessoku

**Affiliations:** 1 Department of Palliative Medicine, International University of Health and Welfare Narita Hospital, 852, Hatakeda, Narita, Chiba 286-8520 Japan; 2 Department of Gastroenterology, International University of Health and Welfare School of Medicine, 4-3, Kozunomori, Narita, Chiba 286-0048 Japan; 3 Department of Gastroenterology and Hepatology, Yokohama City University Graduate School of Medicine, 3-9 Fukuura, Kanazawa-ku, Yokohama, 236-0004 Japan; 4 Department of Medicine and Bioregulatory Science, Graduate School of Medical Sciences, Kyushu University, Fukuoka, 819-0395 Japan; 5 Department of Internal Medicine, University of Medicine and Pharmacy, Ho Chi Minh City, 70000 Vietnam; 6 Department of Nursing, International University Health and Welfare Narita Hospital, 852 Hatakeda, Narita, Chiba 286-8520 Japan; 7 Clinical Laboratory, Kanagawa Dental University Yokohama Clinic, 3-31-6 Turuyacyou, Kanagawa-ku, Yokohama 221-0835 Japan; 8 Deartment of Gastroenterology, Sagami Rinkan Hospital, Sagamihara, 252-0385 Japan; 9 Department of Gastroenterology, Shin-Yurigaoka General Hospital, Kawasaki, 215-0026 Japan; 10 Department of Biostatistics, JORTC Data Center, Tokyo, 116-0013 Japan; 11 Department of Internal Medicine, Iwasaki Naika Clinic, Yokohama, 240-0042 Japan; 12 Department of Internal Medicine, Yokohama Clinic, Kanagawa Dental University, 3-31-6 Tsuruya-cho, Yokohama, Kanagawa 221-0835 Japan; 13 Department of Psychiatry, Chiba Rosai Hospital, Ichihara, 290-0003 Japan; 14 Department of Psychiatry, International University of Health and Welfare Narita Hospital, 852, Hatakeda, Narita, Chiba 286-8520 Japan; 15 Department of Gastroenterology, International University of Health and Welfare Atami Hospital, Shizuoka, 413-0012 Japan

**Keywords:** Stimulant laxatives, Chronic constipation, Laxative dependence, Substance use disorder, Screening

## Abstract

**Background:**

Chronic constipation is common, and the appropriate use of stimulant laxatives remains challenging. Although dependence due to habitual stimulant-laxative use is frequently observed, the long-term effects of regular use on dependence and tolerance have not been fully elucidated. This study aimed to characterize the clinical features and substance use-related symptoms associated with stimulant-laxative use in patients with chronic constipation.

**Methods:**

This multicenter prospective observational study was conducted at three centers and enrolled patients with chronic constipation, including those meeting the Rome IV criteria. Patients were categorized based on stimulant-laxative use into None (*N*), Short-term/Rescue (SR), and Regular (*R*), defined as the use of stimulant laxatives ≥ 3 per week for > 1 year. Substance use–related symptoms were assessed using the CAGE-AID questionnaire (≥ 2 point), and self-reported ICD-11 and DSM-5-TR substance use disorder criteria.

**Results:**

A total of 280 patients were included (*N* = 197, SR = 51, *R* = 32). Baseline characteristics did not differ significantly among groups. CAGE-AID positivity was 5.1%, 58.8%, and 90.6% in the *N*, SR, and *R* groups, respectively. ICD-11 criteria were markedly higher in the *R* group (65.6%) compared with the *N* (0%) and SR (11.8%) groups. DSM-5-TR criteria showed a similar pattern (0%, 15.7%, and 65.6%, respectively). The *R* group consistently demonstrated higher positive screening rates across all tools.

**Conclusions:**

Long-term stimulant-laxative use is associated with increased substance use-related symptoms and features suggestive of habituation in patients with chronic constipation.

**Trial registration:**

The study was registered in the University Hospital Medical Information Network (UMIN000051617) on July 15, 2023.

**Supplementary Information:**

The online version contains supplementary material available at 10.1007/s00535-026-02455-9.

## Introduction

Chronic constipation is a prevalent condition that significantly impairs quality of life (QOL) [1]. Multiple therapeutic options are available, and stimulant laxatives, including bisacodyl, sodium picosulfate, and senna, are widely used due to their established efficacy [2, 3]. However, clinical guidelines recommend limiting stimulant laxatives to intermittent or rescue use [4]. This recommendation is primarily based on reports, suggesting that long-term use may lead to loss of haustral folds and morphological changes in the myenteric plexus of the colon, discouraging use beyond 1 month [5]. In contrast, recent reviews indicate that the evidence supporting harmful morphological changes in the colon due to stimulant laxatives is insufficient [6]. Although the evidence is derived from pediatric populations, long-term use of senna has been reported to carry a risk of blister formation due to prolonged contact between stool and skin, and no evidence of tolerance development has been observed [7]. Consequently, aside from known adverse effects such as electrolyte disturbances secondary to diarrhea or abdominal pain, the basis for limiting stimulant laxatives to intermittent use remains unclear.

In clinical practice, clinicians often encounter patients with persistent constipation who progressively increase stimulant-laxative doses and eventually develop habituation and dependence, making discontinuation difficult. Previous reports on stimulant-laxative misuse have largely focused on their use as a purging method among individuals with eating disorders, particularly anorexia nervosa [8, 9]. However, data on epidemiology, bowel-related QOL, and association with substance dependence among patients with chronic constipation in general outpatient settings remain limited. This gap underscores the need for further investigation. Therefore, this study examined the association between stimulant-laxative use and habituation, substance dependence, and tolerance-related features in patients with chronic constipation.

## Methods

### Ethics and clinical trial registration

This study was approved by the Ethics Committee of the International University of Health and Welfare Narita Hospital (June 27, 2023; Approval No. 23-Nr-009). Additional approvals were obtained from the Ethics Committee of Kanagawa Dental University Yokohama Clinic (Approval No. 957; September 9, 2023) and Iwasaki Internal Medicine Clinic (Approval No. 23-1; August 1, 2023). Written informed consent was obtained from all participants prior to enrollment. The study was registered in the University Hospital Medical Information Network (UMIN000051617; July 15, 2023). All study procedures were conducted in accordance with the ethical principles of the Declaration of Helsinki.

### Study design and participants

This prospective, multicenter observational study was conducted at three medical institutions between July 15, 2023, and September 29, 2024. The lead site was the International University of Health and Welfare Narita Hospital, with Kanagawa Dental University Yokohama Clinic and Iwasaki Internal Medicine Clinic as collaborating centers. Eligible participants were adults (≥ 18 years) with chronic constipation attending outpatient clinics at the participating institutions and who met the Rome IV criteria for functional constipation. Participants were required to undergo colonic transit time (CTT) testing and abdominal ultrasonography and to complete questionnaires and interviews. The study targeted patients with insufficient response to initial constipation management at other healthcare facilities. Exclusion criteria included inability to maintain adequate oral intake, pregnancy or lactation, and communication difficulties.

### Definition of chronic constipation and baseline evaluations

At enrollment, participants completed a baseline interview and self-administered questionnaires detailing demographic characteristics and constipation-related symptoms. Chronic constipation was defined as symptoms for at least 6 months, with at least two of the following occurring for more than 3 months within the preceding 6 months: straining during > 25% of defecations; lumpy or hard stools in > 25% of defecations; sensation of incomplete evacuation, anorectal obstruction, or need for manual maneuvers in > 25% of defecations; or fewer than three spontaneous bowel movements per week. Diagnoses of chronic constipation and defecatory disorders were established by gastroenterology specialists according to Rome IV criteria [10, 11].

Questionnaires assessed age, sex, body mass index (BMI), abdominal circumference, medical history (including abdominal surgery), medication use (dosage and frequency), psychiatric screening items described below, and bowel movement history. Dietary habits were evaluated using two structured questions: the first asked whether the participant’s usual diet was predominantly Western or Japanese, and the second asked whether their primary diet consisted mainly of meat, fish, vegetables, or a balanced combination of these.

### Assessment and classification of stimulant-laxative use

The frequency and types of stimulant laxatives used were assessed through structured interviews. Based on the previous reports [12], regular use was defined as taking stimulant laxatives use ≥ 3 times per week for > 1 year; these patients were categorized as the Regular users (*R*). Patients who reported no stimulant-laxative use were classified as the Non-users (*N*). Those using stimulant laxatives but not meeting regular-use criteria comprised the Short-term/Rescue (SR) group. Participants in the SR and *R* groups reported adverse effects, including abdominal pain and nausea. Use of transanal treatments, including manual disimpaction, enemas, and suppositories, was also assessed through questionnaire-based items.

### Psychiatric screening

Because no established diagnostic criteria exist for dependence on constipation medications, substance use-related characteristics were evaluated using the Cut down, Annoyed, Guilty, Eye-opener-Adapted to Include Drugs (CAGE-AID) questionnaire [13], the International Classification of Diseases, 11th Revision (ICD-11) [14], and the Diagnostic and Statistical Manual of Mental Disorders, Fifth Edition, Text Revision (DSM-5-TR) [15]. CAGE-AID positivity was defined as a score ≥ 2. ICD-11 diagnostic criteria for substance use disorder were applied, including tolerance-related features. DSM-5-TR diagnostic criteria for substance use disorders were used to classify severity as mild (2–3 criteria), moderate (4–5 criteria), or severe (≥ 6 criteria). Depressive symptoms were screened using the Patient Health Questionnaire for Depression Screening (PHQ-2) [16], with a score of ≥ 2 considered positive. Suicidal ideation was assessed using the Japanese version [17] of the Scale for Suicide Ideation (SSI) [18], based on yes/no responses to Question 1 (“Do you currently feel like killing yourself?”) and Question 2 (“Have you ever felt like killing yourself?”).

### Defecation record and QOL analysis

Spontaneous bowel movements (SBMs) were defined as the number of bowel movements per week occurring without rescue medication. Complete spontaneous bowel movements (CSBMs) were defined as SBMs without a sensation of incomplete evacuation, representing higher-quality bowel movements. Stool form was assessed using the Bristol Stool Form Scale (BSFS), which categorizes stool morphology into seven types: Types 1–2 indicate constipation, Types 3–5 indicate normal stool form, and Types 6–7 indicate diarrhea. The proportions of BSFS Type 4 and BSFS Types 3–4 were recorded. Defecatory desire (DD) was evaluated using a five-point Likert scale (1 = always, 2 = often, 3 = sometimes, 4 = rarely, 5 = never). The presence of DD was defined as scores of 1–3, whereas the absence of DD (LODD) was defined as scores of 4–5. Incomplete evacuation and straining were also assessed using a five-point Likert scale; scores of 1–3 indicated presence and 4–5 indicated absence.

Quality of life (QOL) was assessed at baseline using self-administered questionnaires. Satisfaction related to bowel movements was evaluated using a five-point Likert scale (1 = very satisfied, 2 = somewhat satisfied, 3 = neutral, 4 = somewhat dissatisfied, 5 = dissatisfied). Scores of 1–3 were categorized as satisfied, and scores of 4–5 as dissatisfied. Improvements in LODD, straining, incomplete evacuation, and treatment satisfaction were defined as at least a one-point favorable change on the Likert scale from baseline. The transition rate from LODD to DD was defined as the proportion of participants who shifted from LODD (scores 4–5) at baseline to DD (scores 1–3) after treatment. To evaluate overall physical and mental health, the 36-Item Short Form Health Survey (SF-36) [19] was administered. Constipation-specific QOL was assessed using the Japanese version of the Patient Assessment of Constipation Quality of Life (PAC-QOL) [20, 21]. Constipation symptom severity was assessed using the Patient Assessment of Constipation Symptoms (PAC-SYM) [22]. Bowel-related QOL was also assessed using the Constipation Scoring System (CSS) [23, 24].

### Assessment of colon transit time (CTT)

CTT was assessed using radiopaque markers (Sitzmarks^®^, SM). At baseline, all laxatives were discontinued 5 days before SM ingestion, resulting in a 10-day period until abdominal radiography was performed 5 days after ingestion. Abdominal radiographs were obtained 5 days after SM ingestion. CTT measurements from abdominal X-rays were evaluated by radiologic technologists who were blinded to the treatment information. Normal-transit constipation (NTC) and slow-transit constipation (STC) were diagnosed according to the Hinton’s method [25]. STC was defined as the retention of ≥ 20% of markers on abdominal radiography 5 days after SM ingestion, whereas NTC was defined as retention < 20%.

### Observation of rectal ultrasonography (US)

Abdominal ultrasonography (US) was performed using a wireless handheld ultrasound system (FWU-1 [iViz Air]; Fujifilm Corporation, Tokyo, Japan) with a 2–5 MHz convex probe. Rectal ultrasonographic findings on transverse images were classified according to previously published criteria [26] as follows: *R*1 (no stool retention), *R*2 (stool retention), or *R*3 (hard stool). All US examinations were performed by gastroenterology specialists with experience in > 100 bowel ultrasonography procedures.

### Statistical analysis

Statistical analyses were performed using R version 4.4.3 (R Foundation for Statistical Computing, Vienna, Austria). Continuous variables were summarized as means ± standard deviations and medians with interquartile ranges (IQRs), and categorical variables as counts and percentages [*n* (%)]. Missing data were not imputed, and analyses were performed using available data. Welch’s *t* test or Wilcoxon rank-sum test was used for two-group comparisons. For comparisons among the *N*, SR, and *R* groups, both analysis of variance (ANOVA) and the nonparametric Kruskal–Wallis test were used for continuous variables. When ANOVA indicated statistical significance, post hoc multiple comparisons were conducted using *t* tests with Holm correction. When the Kruskal–Wallis test showed significance, pairwise comparisons were performed using the Wilcoxon rank-sum test with Holm correction. Categorical variables were compared using the *χ*^2^ test or Fisher’s exact test when expected cell counts were small. All analyses were two-sided, and statistical significance was defined as *p* < 0.05.

### Propensity score matching

To adjust for baseline imbalances, propensity score matching (PSM) was conducted. The study population was divided into three groups (*N*, SR, and *R*), and independent 1:1 matching was performed for each pairwise comparison (*N* vs SR, *N* vs *R*, and *R* vs SR). Propensity scores were estimated using logistic regression models including age (< 70 vs ≥ 70 years), sex (men vs women), disease duration based on the CSS (< 10 vs ≥ 10 years), and the presence of comorbidities as covariates. Matching was performed using the nearest-neighbor method with a caliper width set at 0.2 times the standard deviation of the logit-transformed propensity score. The discriminative ability of each propensity score model was evaluated using the area under the receiver-operating characteristic curve (AUC). Covariate balance before and after matching was assessed using standardized mean differences (SMDs), with an SMD < 0.1 considered indicative of adequate balance. After matching, between-group comparisons of the proportions of patients who met the ICD-11 tolerance criteria, the DSM-5-TR substance use disorder criteria, and who screened positive on the CAGE-AID questionnaire were performed using McNemar’s test.

## Results

### Demographics

A total of 280 participants were included: 197 in the *N* group, 51 in the SR group, and 32 in the *R* group. Baseline characteristics are shown in Table 1. Age, body weight, BMI, and history of abdominal surgery did not differ significantly among groups. The proportion of female participants was high overall and significantly higher in the SR group than in the other groups. Constipation duration was significantly longer in the SR and *R* groups than in the *N* group, with no significant difference between SR and *R*. No significant between-group differences were observed in medical history or concomitant medications. The proportion of participants consuming meat-based or Western-style diets was significantly lower in the *R* group, with no differences in other dietary patterns. Bowel movement characteristics and rectal ultrasonography findings did not differ significantly among the groups. Regarding colonic transit time, the number of patients with STC was significantly higher in the *R* group. Details of stimulant-laxative use, including duration, types, and transanal treatments, are presented in Supplementary Table S1. Findings from interviews regarding adverse effects of stimulant laxatives are shown in Supplementary Table S2**.**

**Table 1:** Patients demographics

	Stimulant-laxative use category			Overall comparison
	*N*	SR	*R*	
	(*n* = 197)	(*n* = 51)	(*n* = 32)	
Demographics and baseline characteristics				
Age, years, mean (SD)	60 (18)	58 (17)	60 (17)	0.82
Age, years, median (IQR)	65 (50–74)	58 (47–72)	65 (48–72)	0.56
Sex, Female, *n* (%)	136 (69.0)	43 (84.3)	19 (59.4)	0.03
Body weight, kg, mean (SD)	55.8 (11.9)	55.0 (12.2)	55.6 (12.9)	0.92
Body weight, kg, median (IQR)	54.0 (48.0–63.0)	54.0 (46.0–64.0)	58.0 (47.0–67.8)	0.96
Body Mass Index, kg/m^2^, mean (SD)	21.8 (4.2)	21.3 (5.0)	21.4 (3.5)	0.05
Body Mass Index, kg/m^2^, median (IQR)	21.4 (19.5–23.7)	21.6 (19.2–23.6)	20.9 (19.3–24.0)	0.96
History of abdominal surgery, *n* (%)	67 (34.0)	19 (37.3)	13 (40.6)	0.73
Period of illness, years, mean (SD)	18.1 (18.5)	27.8 (18.4)	29.4 (17.0)	0.035
Period of illness, years, median (IQR)	10 (3–32)	30 (10–41)	31 (18–40)	0.013
Comorbidity, *n* (%)				
No comorbidity	21 (10.7)	11 (21.6)	4 (12.5)	0.12
Diabetes mellitus	18 (9.1)	3 (5.9)	5 (15.6)	0.33
Hypothyroidism	4 (2.0)	0 (0.0)	2 (6.2)	0.16
Cerebrospinal disease	14 (7.1)	3 (5.9)	3 (9.4)	0.83
Cardiovascular disease	9 (4.6)	3 (5.9)	3 (9.4)	0.53
Renal dysfunction (including dialysis)	0 (0.0)	0 (0.0)	1 (3.1)	0.02
Psychiatric disorders	10 (5.1)	5 (9.8)	3 (9.4)	0.36
Mood disorders	7 (3.6)	3 (5.9)	2 (6.2)	0.65
Schizophrenia	2 (1.0)	0 (0.0)	1 (3.1)	0.05
Neurodevelopmental disorders	1 (0.5)	1 (2.0)	0 (0.0)	0.48
Eating disorders	0 (0.0)	0 (0.0)	0 (0.0)	NA
Intra-abdominal malignancy	10 (5.1)	1 (2.0)	2 (6.2)	0.58
Extra-abdominal malignancy	4 (2.0)	3 (5.9)	1 (3.1)	0.34
Concomitant medication, *n* (%)				
Concomitant laxatives	65 (33.0)	19 (37.3)	11 (34.4)	0.85
Magnesium oxide	40 (20.3)	10 (19.6)	6 (18.8)	0.98
Polyethylene glycol	8 (4.1)	0 (0.0)	0 (0.0)	0.18
Lactulose	0 (0.0)	0 (0.0)	1 (3.1)	0.02
Lubiprostone	11 (5.6)	2 (3.9)	1 (3.1)	0.78
Linaclotide	6 (3.0)	2 (3.9)	1 (3.1)	0.95
Elobixibat	8 (4.1)	2 (3.9)	1 (3.1)	0.97
Chinese medicine	16 (8.1)	3 (5.9)	4 (12.5)	0.56
Calcium channel blockers use	35 (17.8)	4 (7.8)	4 (12.5)	0.19
Opioid use	1 (0.5)	0 (0.0)	0 (0.0)	0.70
Antidiabetic medication use	16 (8.1)	4 (7.8)	4 (12.5)	0.92
Diuretic use	4 (2.0)	1 (2.0)	1 (3.1)	0.08
Benzodiazepine	10 (5.1)	4 (7.8)	5 (15.6)	0.08
Anticholinergic drugs use (depression related), *n* (%)	9 (4.6)	2 (3.9)	1 (3.1)	1.00
Anticholinergic drugs use (mental/psychiatric), *n* (%)	5 (2.5)	3 (5.9)	2 (6.2)	0.28
Anticholinergic drugs use (BPH), *n* (%)	5 (2.5)	2 (3.9)	3 (9.4)	0.10
Dietary habits, *n* (%)				
Japanese-style meals	120 (60.9)	29 (56.9)	22 (68.8)	0.56
Western-style meals	20 (10.2)	12 (23.5)	1 (3.1)	0.01
Mixed Japanese–Western meals	16 (8.19)	4 (7.8)	4 (12.5)	0.70
Frequent meat intake	31 (15.7)	16 (31.4)	3 (9.4)	0.01
Frequent fish intake	27 (13.7)	7 (13.7)	3 (9.4)	0.92
Frequent vegetable intake	41 (20.8)	8 (15.7)	10 (31.2)	0.24
Balanced intake of meat, fish, and vegetables	97 (49.2)	28 (54.9)	19 (59.4)	0.49
Defecation records				
SBMs, mean (SD), times/week	1.5 (2.3)	1.1 (1.6)	1.3 (1.8)	0.44
SBM, median (IQR), times/week	1.0 (0.0–2.0)	1.0 (0.0–2.0)	0.5 (0.0–2.0)	0.62
CSBM, mean (SD), times/week	0.6 (1.7)	0.4 (0.9)	0.5 (1.0)	0.77
CSBM, median (IQR), times/week	0.0 (0.0–1.0)	0.0 (0.0–0.0)	0.0 (0.0–1.0)	0.29
Loss of defecation desire, *n* (%)	184 (93.4)	43 (84.3)	31 (96.9)	0.08
BSFS, mean (SD), score 1–7	2.0 (1.6)	1.9 (1.5)	2.1 (1.4)	0.76
BSFS, median (IQR), score 1–7	1 (1–2)	1 (1–2)	2 (1.0–2.3)	0.46
BSFS 3 + 4, *n* (%)	21 (10.7)	3 (5.9)	4 (12.5)	0.54
BSFS 4, *n* (%)	10 (5.1)	0 (0.0)	1 (3.1)	0.32
Presence of straining, *n* (%)	168 (85.3)	46 (90.2)	30 (93.8)	0.38
Sense of incomplete evacuation, *n* (%)	118 (59.9)	33 (64.7)	23 (71.9)	0.40
Satisfaction with bowel movement, *n* (%)	5 (2.5)	5 (9.8)	1 (3.1)	0.06
Rectal ultrasonographic findings				
* R*1	126 (64.0)	32 (62.7)	24 (77.4)	0.48
* R*2	49 (24.9)	15 (29.4)	4 (12.5)	
* R*3	22 (11.2)	4 (7.8)	4 (12.5)	
Colon transit time category, *n* (%)				
Normal-transit constipation	166 (84.3)	34 (66.7)	11 (34.4)	< 0.001
Slow transit constipation	31 (15.7)	17 (33.3)	21 (65.6)	

*N* non-stimulant-laxative users, *SR* short-term stimulant-laxative users, *R* regular stimulant-laxative users, *SD* standard deviation, *IQR* interquartile range, *BMI* body mass index, *n* number, *SBM* spontaneous bowel movement, *CSBM* complete spontaneous bowel movement, *BSFS* Bristol Stool Form Scale, *NTC* normal transit constipation, *STC* slow-transit constipation, *BPH* benign prostatic hyperplasia

### Dependence- and tolerance-related features to stimulant laxatives

Awareness of potential adverse effects of stimulant laxatives, including dependence or tolerance, was 9.6% in the *N* group, 74.5% in the SR group, and 84.4% in the *R* group, with the highest rate observed in the *R* group. The proportion of patients who screened positive on the CAGE-AID questionnaire showed a similar trend, being 5.1% in the *N* group, 58.8% in the SR group, and 90.6% in the *R* group. The proportion of patients meeting the ICD-11 diagnostic framework for substance dependence was significantly higher in the *R* group at 65.6% than in the *N* group at 0% and the SR group at 11.8%. The prevalence of patients fulfilling the DSM-5-TR substance use disorder criteria followed the same pattern, with rates of 0% in the *N* group, 15.7% in the SR group, and 65.6% in the *R* group (Supplementary Fig. 1). In addition, based on the ICD-11 tolerance-related feature (item d), tolerance-related criteria items were endorsed in 0% of the *N* group, 11.8% of the SR group, and 65.6% of the *R* group. Similarly, the tolerance-related feature in DSM-5 (item 10) was observed in 0% of the *N* group, 13.7% of the SR group, and 65.6% of the *R* group, with the highest rates in the *R* group (Supplementary Fig. 2). The proportion of patients who screened positive on the PHQ-2 increased progressively from the *N* group to the SR and *R* groups, reaching 40.6% in the *R* group. Regarding suicidal ideation assessed using the SSI, the proportion of patients who responded “yes” to either Question 1 (“Do you currently feel you want to die?”) or Question 2 (“Have you ever felt that you wanted to die?”) was significantly higher in the SR group at 31.4% and the *R* group at 31.2% than in the *N* group at 13.2%, with no significant difference between the SR and *R* groups (Table 2).

**Table 2:** Psychological features and dependence-related measures across stimulant-laxative use groups

Psychological features	Stimulant-laxative use category						
	*N*	SR	*R*		Pairwise comparisons		
	(*n* = 197)	(*n* = 51)	(*n* = 32)	Overall comparison	*N* vs SR	*N* vs *R*	SR vs *R*
Awareness, *n* (%)							
Awareness of dependence/tolerance	19 (9.6)	38 (74.5)	27 (84.4)	< 0.001	< 0.001	< 0.001	0.413
Assessment tools, *n* (%)							
CAGE-AID							
≥ 2 points	10 (5.1)	30 (58.8)	29 (90.6)	< 0.001	< 0.001	< 0.001	< 0.01
Item 1: Cut down	15 (7.6)	35 (68.6)	29 (90.6)	< 0.001	< 0.001	< 0.001	< 0.05
Item 2: Annoyed	0 (0.0)	6 (11.8)	10 (31.2)	< 0.001	< 0.001	< 0.001	< 0.05
Item 3: Guilty	6 (3.0)	14 (27.5)	19 (59.4)	< 0.001	< 0.001	< 0.001	< 0.01
Item 4: Eye opener	10 (5.1)	31 (60.8)	29 (90.6)	< 0.001	< 0.001	< 0.001	< 0.01
ICD-11 positive (≥ 3 points)							
≥ 3 points	0 (0.0)	6 (11.8)	21 (65.6)	< 0.001	< 0.001	< 0.001	< 0.001
Item a: Craving	0 (0.0)	9 (17.6)	21 (65.6)	< 0.001	< 0.001	< 0.001	< 0.001
Item b: Impaired control	0 (0.0)	7 (13.7)	21 (65.6)	< 0.001	< 0.001	< 0.001	< 0.001
Item c: Withdrawal	0 (0.0)	6 (11.8)	21 (65.6)	< 0.001	< 0.001	< 0.001	< 0.001
Item d: Tolerance	0 (0.0)	6 (11.8)	21 (65.6)	< 0.001	< 0.001	< 0.001	< 0.001
Item e: Priority given to use	0 (0.0)	4 (7.8)	19 (59.4)	< 0.001	< 0.01	< 0.001	< 0.001
Item f: Continued use despite harm	0 (0.0)	3 (5.9)	20 (62.5)	< 0.001	< 0.01	< 0.001	< 0.001
DSM-5-TR positive (≥ 2 points)							
≥ 2 points	0 (0.0)	8 (15.7)	21 (65.6)	< 0.001	< 0.001	< 0.001	< 0.001
2–3 points (mild)	0 (0.0)	2 (3.9)	0 (0.0)	0.045	< 0.05	< 0.001	0.52
4–5 points (moderate)	0 (0.0)	0 (0.0)	0 (0.0)	NA	NA	NA	NA
≥ 6 points (severe)	0 (0.0)	6 (11.8)	21 (65.6)	< 0.001	< 0.001	< 0.001	< 0.001
Item 10. Tolerance	0 (0.0)	7 (13.7)	21 (65.6)	< 0.001	< 0.001	< 0.001	< 0.001
Depression screening, *n* (%)							
PHQ-2							
≥ 2 points	37 (18.8)	14 (27.5)	13 (40.6)	0.017	0.178	< 0.01	0.237
Suicide screening, *n* (%)							
SSI							
SSI-Q1 positive (thoughts of self-harm)	4 (2.0)	5 (9.8)	0 (0.0)	0.030	0.020	1.00	0.15
SSI-Q2 positive (wish to die)	26 (13.2)	16 (31.4)	10 (31.2)	< 0.01	0.005	0.016	1.00
SSI-Q1 or Q2 positive (any positive)	26 (13.2)	16 (31.4)	10 (31.2)	< 0.01	0.005	0.016	1.00

*N* non-stimulant-laxative users, *SR* short-term/rescue stimulant-laxative users, *R* regular stimulant-laxative users, *CAGE-AID* Cut down–Annoyed–Guilty–Eye opener-Adapted to Include Drugs, *ICD-11* International Classification of Diseases 11th Revision, *DSM-5-TR* Diagnostic and Statistical Manual of Mental Disorders Fifth Edition Text Revision, *PHQ-2* Patient Health Questionnaire-2, *SSI* Scale for Suicide Ideation, *n* number

### Results of propensity score matching

Baseline characteristics before and after propensity score matching are shown in Supplementary Table S3. The area under the curve of the propensity score models for each pairwise comparison ranged from 0.65 to 0.77, indicating at least moderate discriminative ability. Caliper widths ranged from 0.11 to 0.22. After matching, the numbers of matched patients were 47 per group for *N* vs SR, 31 per group for *N* vs *R*, and 26 per group for *R* vs SR, achieving successful 1:1 matching in all pairs. Before matching, imbalances were observed in several covariates, particularly disease duration and sex in some pairwise comparisons. After matching, covariate balance generally improved, although residual imbalance remained for age, sex, and no comorbidity in the *N* vs *R* comparison and for no comorbidity in the SR vs *R* comparison. Detailed SMDs before and after matching are shown in Supplementary Table S4. In the post-matching analyses, CAGE-AID positivity was significantly higher in the SR group than in the *N* group (57.4% vs 6.4%, *p* < 0.001), as was DSM-5-TR (14.9% vs 0.0%, *p* = 0.023). The ICD-11 positivity was higher in the SR group but did not reach statistical significance (10.6% vs 0.0%, *p* = 0.074). Compared with the *N* group, the *R* group demonstrated significantly higher positivity rates for CAGE-AID (90.3% vs 3.2%, *p* < 0.001), ICD-11 (67.7% vs 0.0%, *p* < 0.001), and DSM-5-TR (67.7% vs 0.0%, *p* < 0.001). In the SR vs *R* comparison, positivity rates for ICD-11 (73.1% vs 19.2%, *p* < 0.01) and DSM-5-TR (73.1% vs 23.1%, *p* < 0.01) were significantly higher in the *R* group, whereas CAGE-AID positivity did not differ significantly between groups (88.5% vs 61.5%, *p* = 0.096) (Table 3).

**Table 3:** Comparison of dependence- and tolerance-related outcomes after propensity score matching

Matched pairs	Outcome	Stimulant-laxative use category		McNemar test
		*N* (%)	SR (%)	
*N* vs SR (*n* = 47)	CAGE-AID positive	3 (6.4)	27 (57.4)	< 0.001
	ICD-11 positive	0 (0.0)	5 (10.6)	0.074
	DSM-5-TR positive	0 (0.0)	7 (14.9)	0.023
*N* vs *R* (*n* = 31)	CAGE-AID positive	1 (3.2)	28 (90.3)	< 0.001
	ICD-11 positive	0 (0.0)	21 (67.7)	< 0.001
	DSM-5-TR positive	0 (0.0)	21 (67.7)	< 0.001
SR vs *R* (*n* = 26)	CAGE-AID positive	16 (61.5)	23 (88.5)	0.096
	ICD-11 positive	5 (19.2)	19 (73.1)	< 0.01
	DSM-5-TR positive	6 (23.1)	19 (73.1)	< 0.01

*N* non-stimulant-laxative users, *SR* short-term/rescue stimulant-laxative users, *R* regular stimulant-laxative users, *ICD-11* International Classification of Diseases 11th Revision, *DSM-5-TR* Diagnostic and Statistical Manual of Mental Disorders Fifth Edition Text Revision, *CAGE-AID* Cut down Annoyed Guilty Eye opener-Adapted to Include Drugs

### Health-related and bowel-related quality of life

For the SF-36, scores for the Physical, Mental, and Role/Social Component Summaries were significantly lower in the *R* group than in the *N* and SR groups, with no significant differences between the *N* and SR groups (Supplementary Fig. 3). Regarding constipation-related QOL measures, the *R* group showed significantly higher (worse) scores than the N and SR groups across all CSS items. Similar trends were observed for JPAC-QOL total and domain scores (Physical discomfort, Psychosocial discomfort, Worries/concerns, and Satisfaction), PAC-SYM total score, and the Abdominal and Stool symptoms domains (Table 4; Supplementary Fig. 4).

**Table 4:** Comparison of constipation-related quality of life and symptom scores across stimulant-laxative use groups

Constipation-related score	Stimulant-laxative use category						
	*N*	SR	*R*		Pairwise comparisons		
	(*n* = 197)	(*n* = 51)	(*n* = 32)	Overall comparison	*N* vs SR	*N* vs *R*	SR vs *R*
Constipation scoring system							
Mean (SD)	11.1 (5.6)	11.7 (5.8)	16.4 (6.7)	< 0.001	0.55	< 0.001	< 0.001
Median (IQR)	10.0 (7.0–14.0)	11.0 (8.5–15.0)	16.0 (11.0–22.1)	< 0.001	0.40	< 0.001	< 0.01
SF-36, mean (SD)							
Physical component summary	47 (10.4)	44.9 (12.5)	37.4 (13.2)	< 0.001	0.24	< 0.001	< 0.01
Mental component summary	47.8 (8.9)	45.3 (10.5)	37.8 (9.3)	< 0.001	0.091	< 0.001	< 0.001
Role/social component summary	48.4 (13.0)	46.8 (16.1)	37.6 (18.0)	< 0.001	0.48	< 0.001	< 0.01
SF-36, median (IQR)							
Physical component summary	49.7 (44.6–53.7)	46.9 (40.9–53.7)	41.2 (27.4–48.7)	< 0.001	0.38	< 0.001	0.028
Mental component summary	49.2 (43.3–52.1)	48.5 (37.6–53.9)	37.4 (27.0–44.1)	< 0.001	0.17	< 0.001	< 0.01
Role/social component summary	55.6 (44.0–56.6)	53.9 (34.9–57.9)	32.5 (25.5–54.4)	< 0.01	0.98	< 0.01	< 0.05
JPAC-QOL, mean (SD)							
Overall	1.7 (0.8)	1.6 (0.8)	2.4 (0.7)	< 0.001	0.77	< 0.001	< 0.001
Physical discomfort	1.6 (1.0)	1.6 (1.0)	2.5 (0.8)	< 0.001	0.97	< 0.001	< 0.001
Psychosocial discomfort	1.2 (0.9)	1.2 (0.9)	1.9 (0.8)	< 0.001	1.00	< 0.001	< 0.01
Worries/concerns	1.6 (1.0)	1.6 (0.9)	2.4 (0.9)	< 0.001	0.81	< 0.001	< 0.001
Satisfaction	2.5 (0.9)	2.4 (0.9)	3.3 (0.5)	< 0.001	0.45	< 0.001	< 0.001
JPAC-QOL, median (IQR)							
Overall	1.5 (0.9–2.3)	1.6 (1.0–2.2)	2.6 (2.1–2.8)	< 0.001	0.82	< 0.001	< 0.001
Physical discomfort	1.5 (0.8–2.5)	1.5 (0.8–2.5)	2.6 (1.9–3.0)	< 0.001	0.94	< 0.001	< 0.001
Psychosocial discomfort	1.0 (0.5–1.8)	1.1 (0.4–1.8)	1.8 (1.3–2.6)	< 0.001	0.90	< 0.001	< 0.01
Worries/concerns	1.6 (0.6–2.4)	1.6 (0.7–2.2)	2.5 (2.2–2.9)	< 0.001	0.97	< 0.001	< 0.001
Satisfaction	2.6 (1.8–3.3)	2.3 (1.8–3.1)	3.1 (2.8–3.8)	< 0.001	0.30	< 0.001	< 0.001
PAC-SYM, mean (SD)							
Overall	1.4 (0.9)	1.5 (0.8)	2.1 (0.8)	< 0.001	0.38	< 0.001	< 0.001
Abdominal symptoms	1.5 (1.1)	1.8 (0.9)	2.7 (0.7)	< 0.001	0.068	< 0.001	< 0.001
Rectal symptoms	0.8 (0.9)	0.7 (0.9)	1.0 (1.3)	0.26	0.65	0.36	0.36
Stool symptoms	1.6 (1.1)	1.7 (1.1)	2.3 (0.9)	< 0.01	0.62	< 0.01	< 0.05
PAC-SYM, median (IQR)							
Overall	1.2 (0.7–1.9)	1.4 (0.8–2.0)	1.8 (1.5–3.1)	< 0.001	0.26	< 0.001	< 0.01
Abdominal symptoms	1.3 (0.8–2.3)	1.8 (1.0–2.3)	2.9 (2.3–3.3)	< 0.001	0.028	< 0.001	< 0.001
Rectal symptoms	0.3 (0.0–1.0)	0.3 (0.0–1.0)	0.7 (0.0–2.3)	0.69	1.0	1.0	1.0
Stool symptoms	1.6 (0.8–2.4)	1.8 (0.9–2.5)	2.2 (1.6–3.1)	< 0.01	0.58	< 0.01	< 0.05

*N* non-stimulant-laxative users, *SR* short-term/rescue stimulant-laxative users, *R* regular stimulant-laxative users, *SD* standard deviation, *IQR* interquartile range, *SF-36* 36-Item Short Form Health Survey, *JPAC-QOL* Japanese version of the Patient Assessment of Constipation Quality of Life, *PAC-SYM* Patient Assessment of Constipation Symptom

## Discussion

This study demonstrated that patients who used stimulant laxatives three or more times per week for over 1 year had significantly higher proportions of positive responses on screening instruments related to substance use symptoms, including CAGE-AID positivity (score ≥ 2), endorsement of ≥ 3 ICD-11 substance use disorder criteria items, and endorsement of ≥ 2 DSM-5-TR substance use disorder criteria items, compared with patients who either did not use stimulant laxatives or used them no more than twice per week. Mental health screening positivity also tended to be higher in regular users using PHQ-2 and SSI. Long-term regular users also exhibited higher proportions of slow-transit constipation, higher positivity rates on depression and suicide-risk screening tools, more severe constipation, and lower defecation-related QOL in comparison with both the N and SR groups. Even after adjusting for baseline characteristics between groups as much as possible, long-term regular stimulant-laxative use remained associated with higher positivity rates for dependence- and tolerance-related measures. Although causality cannot be directly established, these findings suggest that long-term regular stimulant-laxative use may be associated with dependence-related features and tolerance. To our knowledge, no previous studies have comprehensively evaluated epidemiological characteristics or their associations with substance-dependence criteria among chronic users of stimulant laxatives, making this the first report to present such findings.

Although stimulant laxatives are effective, prolonged or excessive use may reduce colonic responsiveness, theoretically contributing to a “cathartic colon,” characterized by loss of haustral folds [12, 27, 28]. While current evidence does not support the notion that stimulant laxatives cause structural colonic damage or increase cancer risk [6], chronic use has been associated with renal impairment and electrolyte abnormalities, including hypokalemia [29, 30]. Bisacodyl and sodium picosulfate are considered effective and safe when used for less than 4 weeks; however, evidence supporting longer-term use remains insufficient, and prolonged use is not recommended [5]. In contrast, senna has been reported to be safe in children, with no evidence of tolerance or major toxicity during long-term use, and concerns regarding cathartic colon associated with long-term senna use lack scientific support [7]. However, evidence for long-term use in adults is lacking, and current U.S. and Japanese guidelines do not recommend chronic use [4, 31]. Therefore, the existing evidence regarding long-term stimulant-laxative use remains limited, and further research on tolerance and safety is warranted.

Misuse of stimulant laxatives is common in clinical settings. Patients with refractory chronic constipation often exhibit excessive preoccupation with bowel movements, poor response to laxatives, and persistently poor clinical course despite repeated consultations [32]. Previous studies on laxative misuse have primarily focused on patients with eating disorders, where laxatives are frequently used as purging behaviors second only to vomiting, and are also common methods of self-harm [33]. Approximately 31% of individuals with eating disorders use laxatives [34], with 67% of those with bulimia nervosa using them for weight control [9], and individuals with anorexia nervosa tending to use laxatives even more frequently [8, 9]. Laxative misuse is associated with depressive tendencies, anorectic behaviors, and symptom severity regardless of diagnostic subtype [35]. It is also strongly linked to borderline personality traits, suicidal ideation, self-injurious behavior, feelings of emptiness, and affective instability [33].

Importantly, laxative misuse is not limited to individuals with eating disorders. It is also observed among athletes requiring strict weight control [36], and among individuals who intentionally induce diarrhea, hypokalemia, and acid–base disorders, abdominal pain, or weight loss through stimulant laxatives [37]. Excessive use is also observed in middle-aged and older adults who escalate laxative use over time [27, 30]. The present study focused on patients without eating disorders and found that long-term stimulant-laxative users had poorer bowel-related QOL, and 84% continued regular use despite awareness of the potential disadvantages. Because laxatives can cause hypokalemia-related cardiotoxicity ranging from mild to severe, occasionally fatal [38], and early psychiatric evaluation should be considered for chronic stimulant-laxative users. These findings may introduce an important new perspective to constipation management.

Patients with constipation often exhibit anxiety, depression, obsessive–compulsive traits, psychotic tendencies, and neurotic characteristics, and show lower emotional stability scores than individuals without constipation [39, 40]. Approximately one-third of refractory constipation cases are independently associated with anxiety, depression, and physical symptoms of constipation [32]. A large cohort study reported that patients with chronic constipation who regularly used laxatives had an increased risk of developing depression (HR 2.33; 95%CI 1.94–2.80) [41], suggesting a strong relationship between refractory constipation and depressive symptoms. Although the prevalence of antidepressant use was low in the study cohort and its impact is, therefore, considered to be limited, the higher prevalence of screening-positive depressive symptoms observed among long-term users may reflect underlying psychological characteristics of refractory constipation. Beyond depressive tendencies, the presence of substance use-related symptoms among long-term users is a novel finding, suggesting an important link between refractory constipation and compulsive or neurotic behavioral tendencies.

This study has several limitations. First, we were unable to differentiate between irritable bowel syndrome, functional constipation, and drug-induced constipation, partly due to the high prevalence of concurrent medication, which made exclusion of drug-induced constipation difficult. Second, because no standardized diagnostic criteria exist for stimulant-laxative dependence, we applied general substance-dependence criteria, which may not fully represent laxative-specific dependence. The CAGE-AID, ICD-11, and DSM-5-TR criteria were applied in this study to capture dependence-like behavioral patterns, rather than diagnose substance use disorders. In addition, PHQ-2 and SSI are screening tools; therefore, these results reflect screening positivity rather than confirmed psychiatric diagnoses. Development of diagnostic criteria for dependence outside psychotropic substances is warranted. Third, although anxiety is strongly associated with constipation severity, we did not include formal anxiety assessments, as this was outside the primary research focus on misuse and dependence. Additionally, formal psychological background assessments were not performed. Therefore, the possibility that unmeasured patient background factors influenced the primary outcomes cannot be completely excluded. Finally, although we screened for histories of eating disorders using medical records, we did not administer standardized screening tools such as the SCOFF questionnaire [42], which should be incorporated in future studies.

In conclusion, chronic, long-term stimulant-laxatives users, defined as individuals using stimulant laxatives three or more times per week for more than 1 year, showed markedly higher rates of meeting screening or diagnostic criteria for dependence and tolerance according to the CAGE-AID questionnaire, ICD-11, and DSM-5-TR, compared with non-regular users. They also demonstrated more severe constipation, poorer defecation-related QOL, and higher prevalence of screening-positive depressive symptoms compared with others. Our findings suggest that limiting stimulant-laxative use to short-term administration may represent a reasonable treatment strategy from the perspective of minimizing dependence- and tolerance-related features. However, further long-term prospective observational studies and interventional trials are warranted to determine whether stimulant laxatives directly contribute to habituation, dependence, tolerance, or delayed colonic transit.

## Supplementary Information

Below is the link to the electronic supplementary material.

Comparison of the positivity rates of CAGE-AID (≥2), DSM-5 (≥2), and ICD-11 (≥3) among N, SR, R groups. Bars represent mean positivity rates, with error bars indicating the standard error of the mean. *p<0.05, **p<0.01, paired t-test for within-group comparisons and Student’s t-test (Welch’s correction when appropriate) for between-group comparisons. Abbreviations: CAGE-AID, Cut-down, Annoyed, Guilty, Eye-opener–Adapted to Include Drugs; DSM-5, Diagnostic and Statistical Manual of Mental Disorders, Fifth Edition; ICD-11, International Classification of Diseases, 11th Revision; N, non-users; SR, short-term/rescue users; R, regular users. Supplementary file1 (TIFF 25495 KB)

Comparison of tolerance-related items based on ICD-11 and DSM-5 among N, SR, and R groups. Bars represent mean positivity rates, with error bars indicating the standard error of the mean. *p<0.05, **p<0.01, paired t-test for within-group comparisons and Student’s t-test (Welch’s correction when appropriate) for between-group comparisons. Abbreviations: DSM-5, Diagnostic and Statistical Manual of Mental Disorders, Fifth Edition; ICD-11, International Classification of Diseases, 11th Revision; N, non-users; SR, short-term/rescue users; R, regular users. Supplementary file2 (TIFF 25495 KB)

Comparison of SF-36 domain scores among N, SR, and R groups. Bars represent mean mean scores, with error bars indicating the standard error of the mean. *p<0.05, **p<0.01, paired t-test for within-group comparisons and Student’s t-test (Welch’s correction when appropriate) for between-group comparisons. Abbreviations: SF-36, 36-Item Short Form Health Survey; PCS, Physical Component Summary; MCS, Mental Component Summary; RCS, Role/Social Component Summary; N, non-users; SR, short-term/rescue users; R, regular users. Supplementary file3 (TIF 3143 KB)

Comparison of JPAC-QOL, PAC-SYM, and CSS scores among N, SR, R groups. Bars represent mean mean scores, with error bars indicating the standard error of the mean. *p<0.05, **p<0.01, paired t-test for within-group comparisons and Student’s t-test (Welch’s correction when appropriate) for between-group comparisons. Abbreviations: JPAC-QOL, Japanese version of the Patient Assessment of Constipation Quality of Life questionnaire; PAC-SYM, Patient Assessment of Constipation Symptoms; CSS, Cleveland Clinic Constipation Score; N, non-users; SR, short-term/rescue users; R, regular users. Supplementary file4 (TIF 2284 KB)

Supplementary file5 (DOCX 24 KB)
