# Supplementary figures and images for: Dependence-related screening positivity among regular stimulant-laxative users with chronic constipation: a multicenter prospective observational study
Source: J Gastroenterol. 2026 May 29;61(8):1123–34. doi: 10.1007/s00535-026-02455-9 (PMC13407722; doi:10.1007/s00535-026-02455-9)

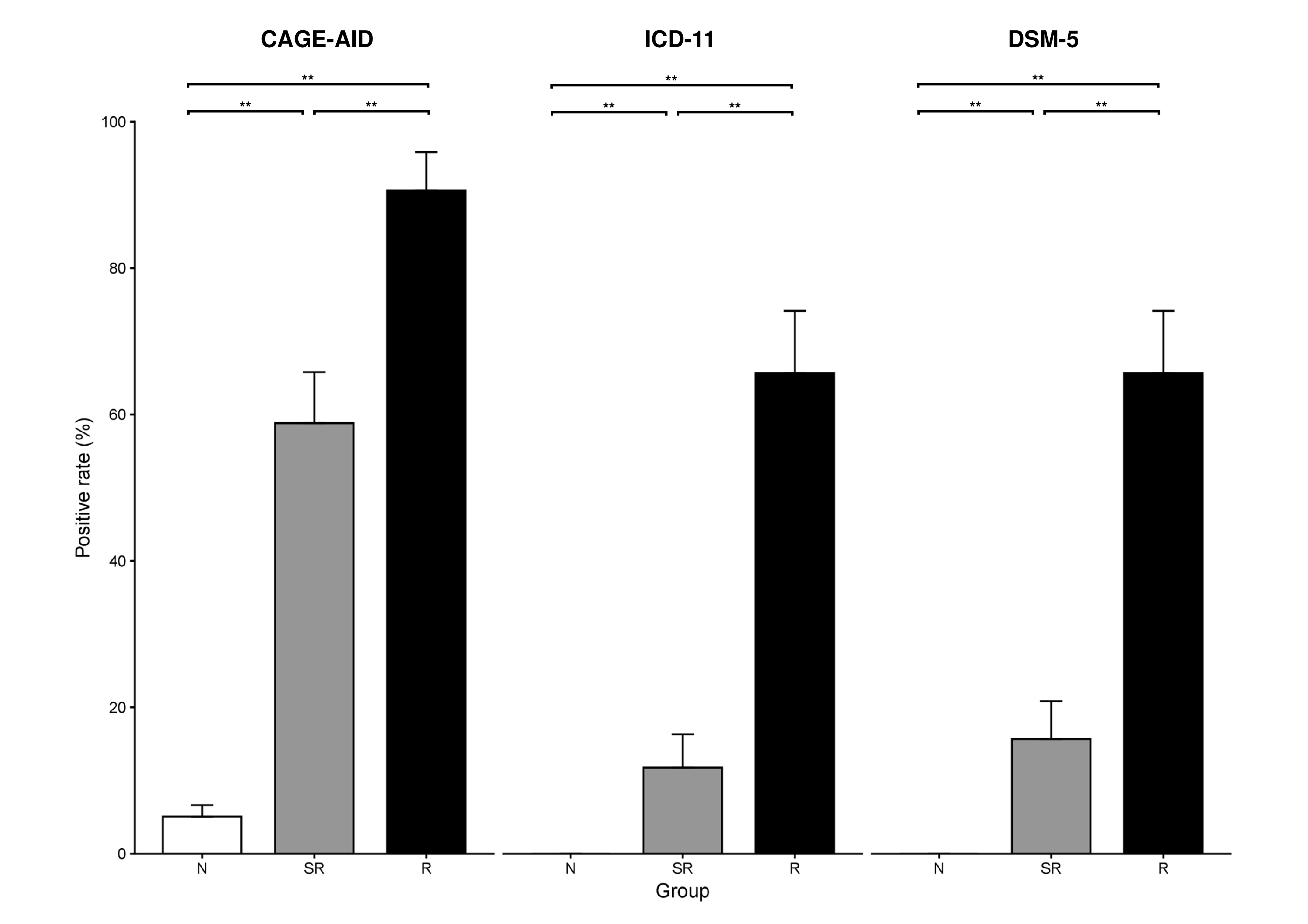

Supplement: Supplementary file 1 — Comparison of the positivity rates of CAGE-AID (≥2), DSM-5 (≥2), and ICD-11 (≥3) among N, SR, R groups. Bars represent mean positivity rates, with error bars indicating the standard error of the mean. *p<0.05, **p<0.01, paired t-test for within-group comparisons and Student’s t-test (Welch’s correction when appropriate) for between-group comparisons. Abbreviations: CAGE-AID, Cut-down, Annoyed, Guilty, Eye-opener–Adapted to Include Drugs; DSM-5, Diagnostic and Statistical Manual of Mental Disorders, Fifth Edition; ICD-11, International Classification of Diseases, 11th Revision; N, non-users; SR, short-term/rescue users; R, regular users. Supplementary file1 (TIFF 25495 KB) [file 535_2026_2455_MOESM1_ESM.tiff]

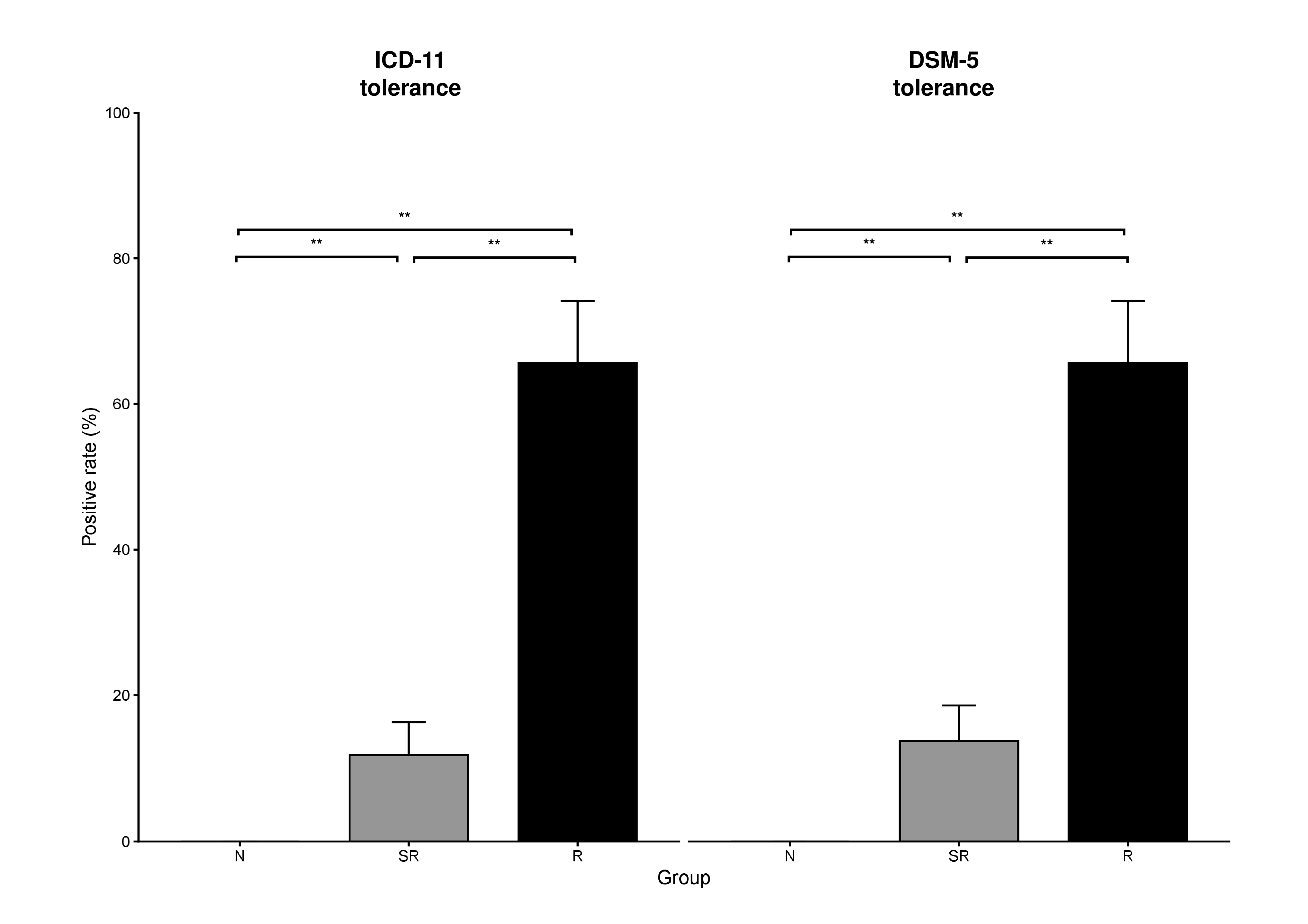

Supplement: Supplementary file 2 — Comparison of tolerance-related items based on ICD-11 and DSM-5 among N, SR, and R groups. Bars represent mean positivity rates, with error bars indicating the standard error of the mean. *p<0.05, **p<0.01, paired t-test for within-group comparisons and Student’s t-test (Welch’s correction when appropriate) for between-group comparisons. Abbreviations: DSM-5, Diagnostic and Statistical Manual of Mental Disorders, Fifth Edition; ICD-11, International Classification of Diseases, 11th Revision; N, non-users; SR, short-term/rescue users; R, regular users. Supplementary file2 (TIFF 25495 KB) [file 535_2026_2455_MOESM2_ESM.tiff]

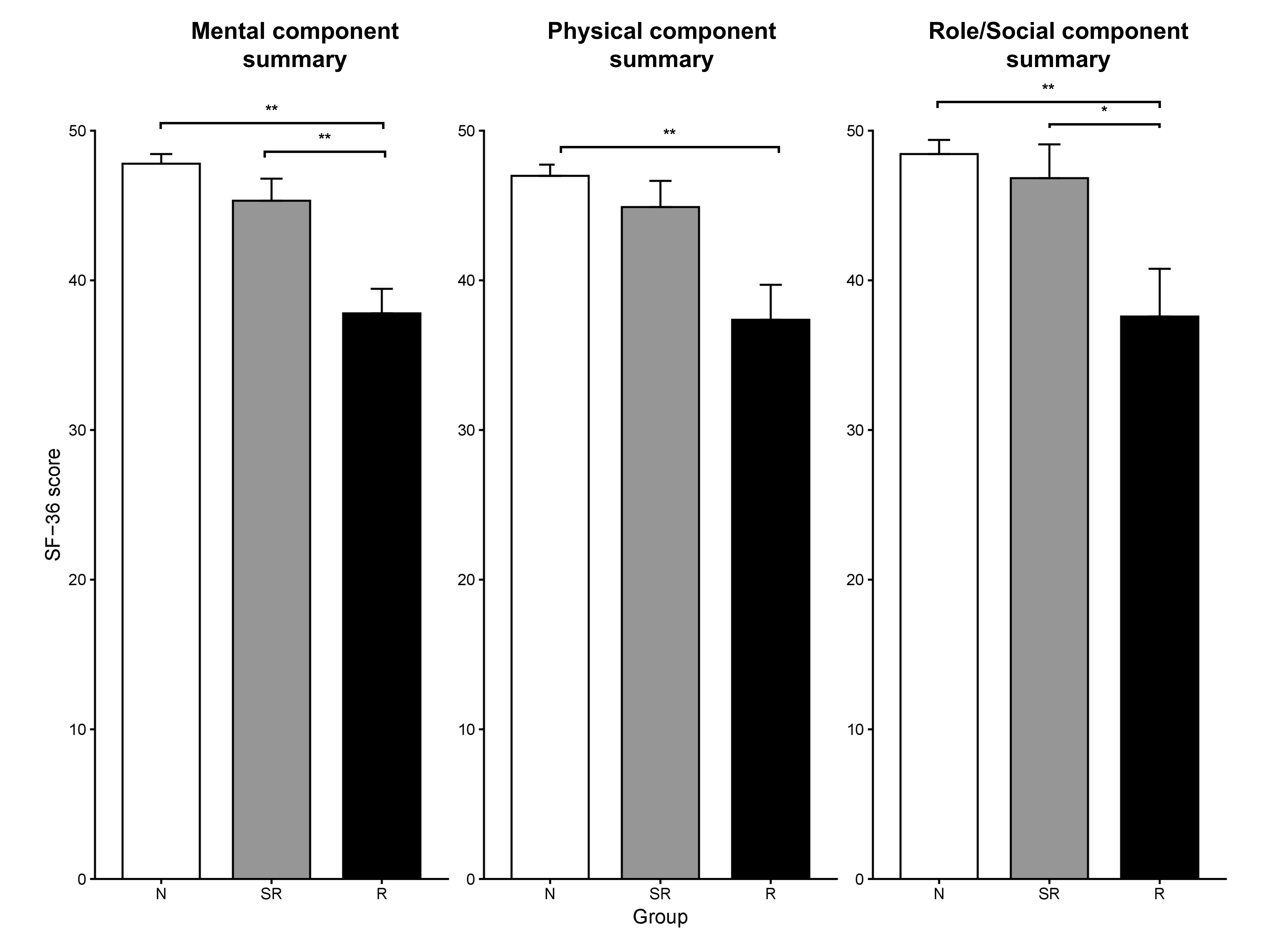

Supplement: Supplementary file 3 — Comparison of SF-36 domain scores among N, SR, and R groups. Bars represent mean mean scores, with error bars indicating the standard error of the mean. *p<0.05, **p<0.01, paired t-test for within-group comparisons and Student’s t-test (Welch’s correction when appropriate) for between-group comparisons. Abbreviations: SF-36, 36-Item Short Form Health Survey; PCS, Physical Component Summary; MCS, Mental Component Summary; RCS, Role/Social Component Summary; N, non-users; SR, short-term/rescue users; R, regular users. Supplementary file3 (TIF 3143 KB) [file 535_2026_2455_MOESM3_ESM.tif]

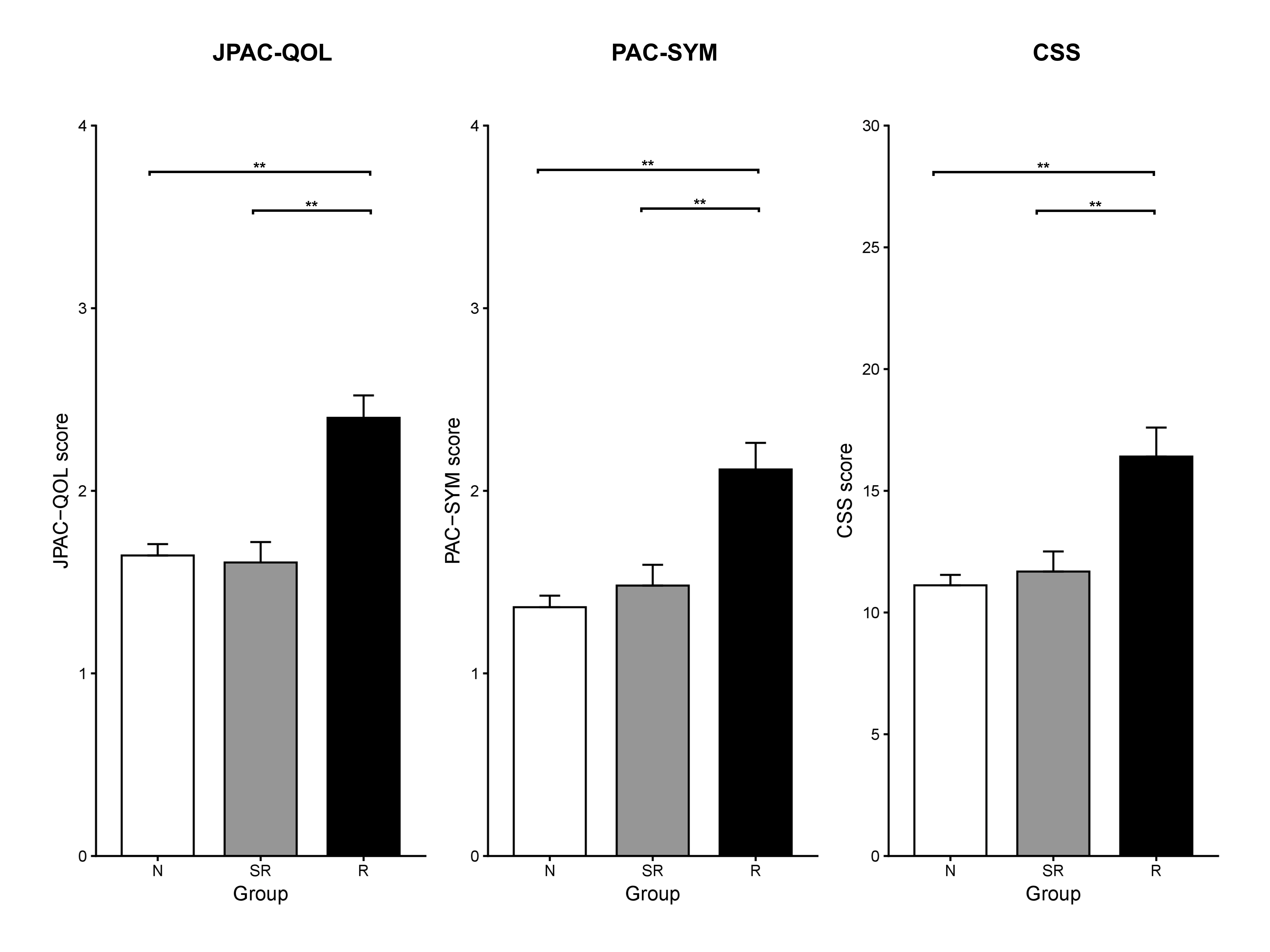

Supplement: Supplementary file 4 — Comparison of JPAC-QOL, PAC-SYM, and CSS scores among N, SR, R groups. Bars represent mean mean scores, with error bars indicating the standard error of the mean. *p<0.05, **p<0.01, paired t-test for within-group comparisons and Student’s t-test (Welch’s correction when appropriate) for between-group comparisons. Abbreviations: JPAC-QOL, Japanese version of the Patient Assessment of Constipation Quality of Life questionnaire; PAC-SYM, Patient Assessment of Constipation Symptoms; CSS, Cleveland Clinic Constipation Score; N, non-users; SR, short-term/rescue users; R, regular users. Supplementary file4 (TIF 2284 KB) [file 535_2026_2455_MOESM4_ESM.tif]
