# Supplementary material for: Dependence-related screening positivity among regular stimulant-laxative users with chronic constipation: a multicenter prospective observational study
Source: J Gastroenterol. 2026 May 29;61(8):1123–34. doi: 10.1007/s00535-026-02455-9 (PMC13407722; doi:10.1007/s00535-026-02455-9)
Supplement: Supplementary file 5 — Supplementary file5 (DOCX 24 KB) [file 535_2026_2455_MOESM5_ESM.docx]

**Supplemental Materials**

**Supplementary Table S1. Detailed Patterns of Stimulant Laxative Use and Additional Transanal Interventions**

|  |  |  | **Stimulant laxative use category** | | |  |  |  |  |
| --- | --- | --- | --- | --- | --- | --- | --- | --- | --- |
|  |  |  | **N** | **SR** | **R** |  | **Pairwise comparisons** | | |
|  |  |  | (n=197) | (n=51) | (n=32) | **Overall comparison** | **N vs SR** | **N vs R** | **SR vs R** |
| **Stimulant Laxative Use, mean (SD)** | | |  |  |  |  |  |  |  |
|  | Duration of use (months) | | 0.0 (0.0) | 74.1 (139.1) | 235.6 (186.9) | <0.001 | <0.001 | <0.001 | <0.001 |
|  | Frequency of use (times) | | 0.0 (0.0) | 372.0 (676.7) | 7067.8 (5606.9) | <0.001 | 0.26 | <0.001 | <0.001 |
| **Stimulant Laxative Use, median (IQR)** | | |  |  |  |  |  |  |  |
|  | Duration of use (months) | | 0.0 (0.0-0.0) | 12.0 (1.0-78.0) | 240.0 (54.0-360.0) | <0.001 | <0.001 | <0.001 | <0.001 |
|  | Frequency of use (times) | | 0.0 (0.0-0.0) | 30.0 (4.0-368.0) | 7200.0 (1620.0-10800.0) | <0.001 | <0.001 | <0.001 | <0.001 |
| **Type of stimulant laxative used, n (%)** | | |  |  |  |  |  |  |  |
|  | None | | 197 (100.0) | 6 (11.8) | 1 (3.1) | <0.001 | <0.001 | <0.001 | 0.24 |
|  | Anthraquinone | | 0 (0.0) | 18 (35.3) | 11 (34.4) | <0.001 | <0.001 | <0.001 | 1.00 |
|  | Diphenylmethane | | 0 (0.0) | 19 (37.3) | 10 (31.2) | <0.001 | <0.001 | <0.001 | 0.64 |
|  | Anthraquinone + Diphenylmethane | | 0 (0.0) | 8 (15.7) | 10 (31.2) | <0.001 | <0.001 | <0.001 | 0.11 |
| **Inappropriate use, n (%)** | | |  |  |  |  |  |  |  |
|  | Yes | | 0 (0.0) | 5 (9.8) | 14 (43.8) | <0.001 | <0.001 | <0.001 | <0.001 |
| **History of transanal treatment, n(%)** | | |  |  |  |  |  |  |  |
|  | None | | 191 (97.0) | 39 (76.5) | 17 (53.1) | <0.001 | <0.001 | <0.001 | 0.033 |
|  | Manual disimpaction | | 0 (0.0) | 1 (2.0) | 0 (0.0) | 0.30 | NA | NA | NA |
|  | Suppository | | 3 (1.5) | 4 (7.8) | 1 (3.1) | <0.05 | 0.10 | 0.91 | 0.91 |
|  | Glycerin enema | | 2 (1.0) | 6 (11.8) | 11 (34.4) | <0.001 | <0.01 | <0.001 | <0.05 |
|  | Suppository + glycerin enema | | 1 (0.5) | 1 (2.0) | 3 (9.4) | <0.01 | 0.37 | <0.01 | 0.29 |
| **Frequency of transanal treatment, n (%)** | | |  |  |  |  |  |  |  |
|  | Rescue or short-term use | | 2 (1.0) | 9 (17.6) | 9 (28.1) | <0.001 | <0.001 | <0.001 | 0.29 |
|  | Daily use | | 0 (0.0) | 1 (2.0) | 4 (12.5) | <0.001 | 0.21 | <0.001 | 0.070 |
| **Volume of glycerin enema, n(%)** | | |  |  |  |  |  |  |  |
|  | 30mL | | 0 (0.0) | 1 (2.0) | 3 (9.4) | 0.0018 | 0.19 | <0.01 | 0.30 |
|  | 40mL | | 0 (0.0) | 0 (0.0) | 1 (3.1) | 0.11 | NA | NA | NA |
|  | 60mL | | 3 (1.5) | 4 (7.8) | 10 (31.2) | <0.001 | 0.026 | <0.001 | 0.014 |
|  | 120mL | | 0 (0.0) | 1 (2.0) | 0 (0.0) | 0.28 | NA | NA | NA |
|  | 150mL | | 0 (0.0) | 1 (2.0) | 0 (0.0) | 0.28 | NA | NA | NA |
| **Willingness surgery for constipation, n(%)** | | | |  |  |  |  |  |  |
|  | Yes | | 1 (0.5) | 0 (0.0) | 10 (31.2) | <0.001 | 1.00 | <0.001 | <0.001 |

*Abbreviations: N, non-stimulant-laxative users; SR, short-term/rescue stimulant-laxative users; R, regular stimulant-laxative users;* *SD, standard deviation; IQR, interquartile range.*

**Supplementary Table S2. Characteristics of abdominal pain and related symptoms among patients using stimulant laxatives**

|  |  |  |  | **Stimulant laxative use category** | |  |
| --- | --- | --- | --- | --- | --- | --- |
|  |  |  | **Overall** | **Short term + Rescue  (SR)** | **Regular**  **(R)** | **SR vs R** |
| **Clinical characteristics of abdominal pain** | | | (n=83) | (n=51) | (n=32) | *p* value |
| **Abdominal pain** | | |  |  |  |  |
|  | **Site, n (%)** | |  |  |  |  |
|  |  | Right hypochondriac region | 13 | 8 (15.7) | 5 (15.6) | 1.00 |
|  |  | Epigastric region | 12 | 9 (17.6) | 3 (9.4) | 0.35 |
|  |  | Left hypochondriac region | 11 | 8 (15.7) | 3 (9.4) | 0.52 |
|  |  | Right lumbar region | 23 | 18 (35.3) | 5 (15.6) | 0.078 |
|  |  | Umbilical region | 18 | 14 (27.5) | 4 (12.5) | 0.17 |
|  |  | Left lumbar region | 18 | 14 (27.5) | 4 (12.5) | 0.17 |
|  |  | Right iliac region | 25 | 18 (35.3) | 7 (21.9) | 0.23 |
|  |  | Hypogastric region | 14 | 11 (21.6) | 3 (9.4) | 0.23 |
|  |  | Left iliac region | 14 | 11 (21.6) | 3 (9.4) | 0.23 |
|  |  | Whole abdomen | 11 | 8 (15.7) | 3 (9.4) | 0.52 |
|  | **Severity** | |  |  |  |  |
|  |  | Numerical rating scale (NRS), mean (SD) | 6.6 (2.5) | 6.6 (2.6) | 6.4 (2.3) | 0.85 |
|  |  | Numerical rating scale (NRS), median (IQR) | 6.5 (4.3-9.0) | 6.0 (5.0-9.0) | 7.0 (4.0-8.0) | 0.78 |
|  |  | Intolerable pain, n (%) | 24 (28.9) | 20 (39.2) | 4 (12.5) | 0.40 |
|  | **Time course** | |  |  |  |  |
|  |  | Time to abdominal pain onset, hours: mean (SD) | 6.5 (2.8) | 6.2 (2.7) | 7.1 (3.7) | 0.52 |
|  |  | Duration of abdominal pain, hours: mean (SD) | 3.1 (4.0) | 3.5 (4.8) | 1.7 (1.2) | 0.085 |
|  |  | Time to abdominal pain onset, hours: median (IQR) | 6.0 (5.0-8.0) | 6 (5-8) | 7 (3-10) | 0.52 |
|  |  | Duration of abdominal pain, hours: median (IQR) | 2.0 (1.0-3.0) | 2.0 (1.0-3.5) | 1.0 (0.8-3.0) | 0.30 |
|  | **Type, n (%)** | |  |  |  |  |
|  |  | Cramping pain | 3 (3.6) | 1 (2.0) | 2 (6.2) | 0.091 |
|  |  | Pressing pain | 26 (31.3) | 20 (39.2) | 6 (18.8) | <0.001 |
|  |  | Throbbing pain | 8 (9.6) | 7 (13.7) | 1 (3.1) | <0.001 |
|  |  | Colicky pain | 2 (2.4) | 2 (3.9) | 0 (0.0) | <0.01 |
| **Associated symptoms, n (%)** | | |  |  |  |  |
|  |  | Nausea | 10 (12.0) | 6 (11.8) | 4 (12.5) | 1.00 |
|  |  | Dizziness | 4 (4.8) | 4 87.8) | 0 (0.0) | 0.16 |
|  |  | Flushing | 2 (2.4) | 1 (2.0) | 1 (3.1) | 1.00 |
|  |  | Syncope | 1 (1.2) | 1 (2.0) | 0 (0.0) | 1.00 |
|  |  | Diaphoresis | 1 (1.2) | 1 (2.0) | 0 (0.0) | 1.00 |
| **Effectiveness of stimulant laxatives, n (%)** | | |  |  |  |  |
|  |  | Yes | 32 (38.6) | 24 (47.1) | 8 (25.0) | 0.064 |

*Abbreviations: N, non-stimulant-laxative users; SR, short-term/rescue stimulant-laxative users; R, regular stimulant-laxative users;* *SD, standard deviation; IQR, interquartile range.*

**Supplementary Table S3. Baseline characteristics before and after propensity score matching**

|  | Group | Matching status | n | Age ≥70 years, n (%) | Women, n (%) | Illness period ≥11 years, n (%) | No comorbidity, n (%) |
| --- | --- | --- | --- | --- | --- | --- | --- |
| N vs SR (n = 47) | N | Before | 168 | 69 (41.1) | 120 (71.4) | 34 (20.2) | 21 (12.5) |
|  | N | After | 47 | 14 (29.8) | 40 (85.1) | 16 (34.0) | 9 (19.1) |
|  | SR | Before | 49 | 15 (30.6) | 42 (85.7) | 19 (38.8) | 11 (22.4) |
|  | SR | After | 47 | 15 (31.9) | 40 (85.1) | 17 (36.2) | 9 (19.1) |
| N vs R (n = 31) | N | Before | 168 | 69 (41.1) | 120 (71.4) | 34 (20.2) | 21 (12.5) |
|  | N | After | 31 | 7 (22.6) | 24 (77.4) | 20 (64.5) | 2 (6.5) |
|  | R | Before | 32 | 10 (31.2) | 19 (59.4) | 21 (65.6) | 4 (12.5) |
|  | R | After | 31 | 10 (32.3) | 19 (61.3%) | 20 (64.5) | 4 (12.9) |
| SR vs R (n = 26) | SR | Before | 49 | 15 (30.6) | 42 (85.7) | 19 (38.8) | 11 (22.4) |
|  | SR | After | 26 | 5 (19.2) | 19 (73.1) | 14 (53.8) | 3 (11.5) |
|  | R | Before | 32 | 10 (31.2) | 19 (59.4) | 21 (65.6) | 4 (12.5) |
|  | R | After | 26 | 6 (23.1) | 19 (73.1) | 15 (57.7) | 4 (15.4) |

*Abbreviations: N, non-stimulant-laxative users; SR, short-term/rescue stimulant-laxative users; R, regular stimulant-laxative users.*

**Supplementary Table S4. Covariate balance before and after propensity score matching**

| Comparison | Covariate | SMD before matching | SMD after matching |
| --- | --- | --- | --- |
| N vs SR | Age ≥70 years | 0.227 | 0.046 |
|  | Women | 0.408 | 0.000 |
|  | Illness period ≥11 years | 0.380 | 0.044 |
|  | No comorbidity | 0.238 | 0.000 |
| N vs R | Age ≥70 years | 0.212 | 0.209 |
|  | Women | 0.245 | 0.328 |
|  | Illness period ≥11 years | 0.956 | 0.000 |
|  | No comorbidity | 0.000 | 0.195 |
| SR vs R | Age ≥70 years | 0.014 | 0.083 |
|  | Women | 0.536 | 0.000 |
|  | Illness period ≥11 years | 0.565 | 0.081 |
|  | No comorbidity | 0.301 | 0.116 |

*Abbreviations: SMD, standardized mean difference. Absolute SMD values are shown.*
